# Hydrothermal Pretreatment of Lignocellulosic Feedstocks to Facilitate Biochemical Conversion

**DOI:** 10.3389/fbioe.2022.846592

**Published:** 2022-02-16

**Authors:** Carlos Martín, Pooja Dixit, Forough Momayez, Leif J. Jönsson

**Affiliations:** ^1^ Department of Chemistry, Umeå University, Umeå, Sweden; ^2^ Department of Biotechnology, Inland Norway University of Applied Sciences, Hamar, Norway

**Keywords:** hydrothermal pretreatment, lignocellulose, enzymatic saccharification, biochemical conversion, sugar-platform process

## Abstract

Biochemical conversion of lignocellulosic feedstocks to advanced biofuels and other bio-based commodities typically includes physical diminution, hydrothermal pretreatment, enzymatic saccharification, and valorization of sugars and hydrolysis lignin. This approach is also known as a sugar-platform process. The goal of the pretreatment is to facilitate the ensuing enzymatic saccharification of cellulose, which is otherwise impractical due to the recalcitrance of lignocellulosic feedstocks. This review focuses on hydrothermal pretreatment in comparison to alternative pretreatment methods, biomass properties and recalcitrance, reaction conditions and chemistry of hydrothermal pretreatment, methodology for characterization of pretreatment processes and pretreated materials, and how pretreatment affects subsequent process steps, such as enzymatic saccharification and microbial fermentation. Biochemical conversion based on hydrothermal pretreatment of lignocellulosic feedstocks has emerged as a technology of high industrial relevance and as an area where advances in modern industrial biotechnology become useful for reducing environmental problems and the dependence on fossil resources.

## Introduction

The negative environmental impact of the extensive use of fossil fuels and problems associated with the dependency on fossil resources to produce energy, chemicals, and materials have strengthened efforts devoted to increased utilization of renewable feedstocks (Weinberg and Kaltschmitt, 2013). Lignocellulosic biomass is formed at a high rate (∼200 × 10^9^ tons per year), is relatively inexpensive, and has large potential as feedstock for sustainable production of biofuels, platform chemicals, and value-added products (Bhowmick et al., 2018).

Lignocellulosic biomass includes agricultural and agro-industrial residues, forest and wood-processing residues, herbaceous energy crops and short-rotation trees, and a part of municipal solid waste. An overview of the composition of different lignocellulosic materials, including softwood (pine), hardwood (eucalyptus), an agro-industrial by-product (sugarcane bagasse), and two agricultural residues (wheat straw and corn stover), is given in Table 1. The main organic constituents of lignocellulosic biomass are cellulose, hemicelluloses, and lignin, which are closely associated in lignin-carbohydrate complexes (LCC) in the secondary cell walls of vascular plants (Fengel and Wegener, 1989). The cellulose content is comparable in most lignocellulosic materials, while the contents of hemicelluloses and lignin differ greatly in different types of biomass. Besides water, other constituents include extractives and minerals. The distribution of extractives and minerals varies greatly depending on the type of biomass (Table 1).

**TABLE 1:** Summary of the composition of different types of lignocellulosic biomass (mass fraction in percent dry weight).

Biomass	Cellulose	Hemi-celluloses	Lignin	Extractives	Minerals	References
Maritime pine (*Pinus pinaster*)	45.0	22.2^a^	26.8^b^	2.9	0.2	López et al. (2020)
Eucalyptus (*Eucalyptus nitens*)	42.0	22.2^c^	22.9	4.7	0.3	Penín et al. (2019)
Sugarcane (*Saccharum officinaru*m) bagasse	36.9	24.5	22.0	4.7	4.5	Neves et al. (2016)
Wheat (*Triticum aestivum*) straw	34.0	22.9^c^	15.0	14.8	4.3	Cornejo et al. (2019)
Corn (*Zea mays*) stover	38.2	25.8^c^	17.4	13.3	5.3	Li and Kim, (2011)

a Mainly hexosans.

b Klason lignin.

c Mainly pentosans.

Plant cellulose is a linear polysaccharide composed of glucose units linked by β-1,4-glycosidic bonds. The degree of polymerization (DP) reaches as high as 15,000 (Fengel and Wegener, 1989). Cellulose chains are packed in a compact crystalline structure stabilized by hydrogen bonds and hydrophobic interactions (Lindman et al., 2021). Crystallinity contributes to giving cellulose a low reactivity towards chemicals and enzymes. There are also amorphous regions, which are more prone to chemical and enzymatic reactions, but they are a minor part of the macromolecule.

Hemicelluloses are branched heteropolysaccharides composed of units of pentoses, hexoses, and uronic acids. They have relatively low DP (Fengel and Wegener, 1989). Softwood hemicelluloses are rich in hexose units, while pentose units are prevalent in hardwood hemicelluloses. *O*-Acetyl-galactoglucomannan and *O*-acetyl-4-*O*-methylglucurono-D-xylan are the main hemicelluloses in softwood and hardwood, respectively. In gramineous plants, which are the main source of agricultural and agro-industrial residues, hemicelluloses are predominantly pentosans. Hemicelluloses are amorphous and have higher reactivity than cellulose. They can undergo hydrolysis under relatively mild conditions, which is of crucial importance in lignocellulose biorefining.

Lignin is a polymer consisting of phenylpropane units, which are linked by ether and carbon-carbon bonds and which form a three-dimensional network (Fengel and Wegener, 1989; Ralph et al., 2019). The β-O-4 ether linkage is the most common intermonomeric linkage in lignin. The lignin content is typically higher in wood than in gramineous biomass, and is especially high in softwood (Table 1). Lignin is primarily composed of three types of phenylpropanoid units, i.e., guaiacyl (G), syringyl (S), and *p*-hydroxyphenyl (H). These units are derived from three different monolignols: G units from coniferyl alcohol, S units from sinapyl alcohol, and H units from *p*-coumaryl alcohol. Softwood lignin consists almost exclusively of G units, while hardwood lignin is a mixture of G and S units. In gramineous lignin, H units are also important constituents, besides G and S. Although less prominent than G, S, and H units, other types of units sometimes occur, such as cinnamyl alcohol end groups, and *p*-hydroxybenzoate and *p*-coumarate conjugates (Ralph et al., 2019). Since the association of lignin with polysaccharides in LCC makes enzymatic access to cellulose difficult, removal of lignin is beneficial for enzymatic saccharification.

In a lignocellulose biorefinery, hemicelluloses, lignin, and cellulose are fractionated into streams that are then valorized to fuels, chemicals, and materials. Carbohydrates can be processed to biofuels (such as ethanol and butanol) and to platform chemicals (such as furans and organic acids). Lignin can be converted to phenols, polymers, composites and different added-value specialty chemicals and fuels. Lignocellulose biorefining can be based on different fractionation sequences. One approach is to first separate hemicelluloses, and then submit the resulting cellulignin to further fractionation by either lignin solubilization or cellulose saccharification. Another approach is to target the lignin, which is an approach that has been used for a long time in chemical pulping processes (the Kraft process, the sulfite process, the soda process, and the organosolv process). More recently, this fractionation approach has become known as the lignin-first approach (Matsakas et al., 2019).

Although the first mention of the word “biorefinery” dates from the early 1990s (Wyman and Goodman, 1993), industrial biorefining has preceded the usage of the term. The fundaments of biorefining were set by the pulp and paper industry for more than one century ago (Alén, 2015), and since then it has for a long time produced a variety of bio-based commodities beyond pulp and paper. Cane sugar mills serve as another example of proto-biorefineries. Sugarcane bagasse, molasses, and filter cakes have for a long time been used for energy generation, ethanol production, and wax extraction, respectively. Modern pulp mills performing green manufacturing of several end-products from one feedstock are sophisticated biorefineries (Alén, 2015). The same applies to state-of-the-art sugarcane-processing complexes, where production of food, biofuel, chemicals, electricity, and heat is integrated at the same production plant (Vaz, 2019).

The term “pretreatment” is used in different areas for referring to operations that are performed prior to certain major processes in order to improve their performance. In this paper, pretreatment refers to processing of lignocellulosic biomass to facilitate enzymatic saccharification of cellulose. Pretreatment and enzymatic saccharification, followed by microbial fermentation or chemical conversion of sugars and valorization of lignin, are parts of the sugar-platform route, which aims at producing advanced biofuels and other bio-based products from lignocellulose (Wyman and Dale, 2015).

Enzymatic saccharification of raw lignocellulose would result in low rates and yields due to feedstock recalcitrance, a set of properties that obstruct the access of enzymes to cellulose. Pretreatment is required to reduce the recalcitrance and thereby facilitate enzymatic saccharification. Pretreatment would typically affect both the structure and the chemistry of the biomass (Zhao et al., 2012a). An effective pretreatment should result in greatly enhanced enzymatic digestibility of cellulose and in high recovery of hemicellulosic saccharides (Jönsson and Martín, 2016). Many pretreatment approaches have been developed as a result of intense research in the area, and some of the most effective methods have been validated at demonstration scale (Galbe and Wallberg, 2019). Research on different raw materials has shown that the effectiveness of pretreatment is feedstock-dependent, and that the effects of a given method can diverge for different types of lignocellulosic biomass (Martín, 2021). Low capital expenditures (capex) and operational expenditures (opex) are key criteria for industrially viable pretreatment methods. In that sense, methods allowing operation at high-solids loadings and with low use of expensive chemical additives are relevant for upscaling. Demonstration-scale operations have allowed the gathering of engineering information needed for full-scale design, and some technically relevant methods have been upscaled to commercial plants (Table 2).

**TABLE 2:** Examples of hydrothermal pretreatment approaches and other commercially relevant methods.

Method	Effects on lignocellulose constituents	Upscaling examples
Auto-catalyzed hydrothermal pretreatment	Partial solubilization of hemicelluloses, slight effects on cellulose and lignin	Inbicon, RE Energy (Denmark), Clariant (Switzerland)
Hydrothermal pretreatment with dilute acid	Extensive hydrolysis of hemicelluloses, hydrolysis of amorphous cellulose, minor fragmentation of lignin	Iogen Corporation (Canada), POET-DSM (United States ), Raízen Energia (Brazil)
Hydrothermal pretreatment with steam explosion	Partial to complete solubilization of hemicelluloses, fragmentation of cellulose, minor fragmentation of lignin	Sekab (Sweden), Abengoa Bioenergy (United States )
Mild alkaline methods	Significant removal of lignin, partial solubilization of hemicelluloses, deacetylation	DuPont (United States )
Chemical pulping- processes (including sulfite and organosolv)	Extensive removal of lignin, variable removal of hemicelluloses, decrease of degree of polymerization and crystallinity of cellulose	Borregaard (BALI process) (Norway), Chempolis (Finland)

Different varieties of hydrothermal pretreatment (HTP) and some processes originating from the pulping industry are among the methods of higher technical relevance (Table 2). In HTP, moist feedstocks, either alone or in the presence of chemical additives, are subjected to high temperature for a certain period of time. HTP is generally performed at acidic pH, which is caused either by organic acids released from the biomass or by added acids. In HTP, especially when a low initial pH is applied, hemicelluloses are hydrolyzed, while most of the cellulose and lignin will remain in the pretreated solid biomass. Removal of hemicelluloses increases biomass surface area, and results in an improvement of the enzymatic digestibility of cellulose.

The sulfite-based BALI (short for “Borregaard Advanced Lignin”) process of Borregaard (Table 2) (Rødsrud et al., 2012) and organosolv pretreatment are technically-relevant methods that emerged from the pulping industry. In contrast to HTP under acidic conditions, chemical-pulping-based methods follow the lignin-first philosophy, i.e., lignin is the main target. Hemicelluloses are also solubilized and can potentially be separated from lignin before upgrading. Cellulose remains in the solid fraction, and its susceptibility to enzymatic saccharification is greatly enhanced. Other pretreatment methods than HTP are, however, beyond the scope of this review, and more detailed information about them can be found elsewhere (Jönsson and Martín, 2016; Galbe and Wallberg, 2019).

In hydrothermal processing biomass is treated in the presence of water at temperatures in the order ∼200–400°C and at high pressure. Although hydrothermal processing includes hydrothermal (HT) liquefaction, HT gasification, and HT carbonization (Tekin et al., 2014), this review focuses on hydrothermal pretreatment (HTP) of lignocellulosic biomass prior to enzymatic saccharification of cellulose.

HTP is one of the most technologically-mature pretreatment methods for lignocellulosic feedstocks (Ruiz et al., 2020), and it is used in many biorefinery upscaling attempts. The simplest HTP procedure is auto-catalyzed hydrothermal pretreatment (A-HTP), which includes only disintegrated biomass, water, and heating to temperatures typically in the range ∼150–230°C. A major strength of A-HTP is that neither chemical additives (except water and alkali for subsequent pH adjustments), nor expensive anticorrosion materials are required, which lowers capex. Furthermore, A-HTP can be performed continuously and with high-solids loading, which is convenient for industrial operation. An A-HTP process was validated for wheat straw at the Inbicon demonstration plant in Kalundborg, Denmark (Larsen et al., 2012). Commercial-scale initiatives based on A-HTP include, e.g., the plant of Clariant in Podari, Romania (Hortsch and Corvo, 2020) and RE Energy (BEST, 2021).

In HTP, temperature and residence time can be modified to modulate the severity of the pretreatment. HTP severity can be estimated by using the severity factor (SF), an equation combining time and temperature into a single variable (Chornet and Overend, 2017). The severity concept can also cover acidity, which is included in the combined severity factor (CSF). HTP effectiveness for different biomass materials can be optimized by tuning SF or CSF.

The effects of HTP can be potentiated by either including an explosion at the end of the holding period or running the process at starting pH values far from neutrality. In hydrothermal pretreatment with steam explosion (HTP-SE), a sudden decompression is applied after steaming the biomass in a closed chamber. This results in mechanical disruption of the material. Low starting pH, achieved by adding an acid, is typical for dilute-acid-catalyzed hydrothermal pretreatment (DA-HTP), commonly known as dilute-acid pretreatment. DA-HTP is also often combined with steam explosion. Treatment at higher pH (Kim et al., 2016), such as mild alkaline conditions, can be considered as another type of HTP approach.

Depending on the pH of the pretreatment, the composition of the lignocellulose will change dramatically (Galbe and Wallberg, 2019). At acidic pH, the main effect is hydrolysis of hemicelluloses, often all the way to monosaccharides. Alkaline pH promotes the dissolution of lignin, whereas cellulose and a part of the hemicelluloses remain in the solid fraction (Table 2). In A-HTP, where the pH becomes acidic after a short while, hemicelluloses are hydrolyzed to a lesser extent than in DA-HTP, but to a greater extent than under mild alkaline conditions. Lignin is fragmented by cleavage of some of the β-O-4 linkages, but most of it is not dissolved as under strong alkaline conditions. A reason for that is that alkaline conditions cause deprotonation of phenolic hydroxyl groups in lignin to phenolate ions, which facilitate dissolution of lignin in aqueous medium. Compared to hydrothermal pretreatment under mild alkaline conditions, DA-HTP requires higher temperatures, and compared to A-HTP, it requires corrosion-resistant alloys and typically leads to more by-product formation. Nevertheless, due to its effectiveness and ease of implementation, DA-HTP is the option of choice in many upscaling initiatives (Solarte-Toro et al., 2019) (Table 2). HTP-SE requires energy for reaching the required temperatures and pressures and also sophisticated equipment, but that is counterbalanced by its effectiveness towards different feedstocks, and, therefore, it is also attractive for commercial applications (BEST, 2021).

HTP can be discussed in a narrow sense and, as in this review, in a broad sense. In a more narrow sense, it is restricted to pretreatment of biomass in hot water or steam, as in A-HTP. However, due to auto-catalysis and formation of carboxylic acids that acidify the reaction mixture, A-HTP is an acidic pretreatment process and final pH values in the range 2-3 are commonly observed. In a broader sense, HTP also includes pretreatment techniques in which small amounts of acid or alkali are added to the reaction mixture, and certain variations that can be used to enhance the effect of hydrothermal pretreatment, such as HTP-SE and HTP with addition of gas (Ilanidis et al., 2021a). As higher temperature and/or longer residence time leads to formation of more carboxylic acids and results in lower pH, the differences between A-HTP under harsh conditions and DA-HTP under mild conditions become small. Furthermore, the same equipment is typically used for both A-HTP and DA-HTP, and the choice between them is governed mainly by which type of biomass that is pretreated (as wood, and particularly softwood, typically requires harsher conditions than gramineous plants). These aspects motivate a discussion of HTP in the broader sense.

## Hydrothermal Pretreatment for Biochemical Conversion

### Hydrothermal Pretreatment

Hydrolysis of hemicelluloses, a crucial aspect of HTP, follows the classical mechanism of acid hydrolysis of polysaccharides, i.e., splitting of glycosidic linkages is catalyzed by a proton transferred from an acid catalyst to the glycosidic oxygen atom, followed by water addition to the anomeric carbon (Loerbroks et al., 2015). The simplest catalysis approach in HTP is auto-catalysis, typical for A-HTP, which is also known as auto-hydrolysis. In A-HTP, heating of moist biomass causes water auto-ionization, and the formed hydronium ions catalyze xylan deacetylation. Dissociation of resulting acetic acid provides protons that push partial hydrolysis of hemicelluloses (Ruiz et al., 2020). Uronic acids released from hemicelluloses also contribute to the catalysis. Hydrolysis proceeds deeper in DA-HTP, where the catalysis is reinforced by inclusion of an acid in the reaction mixture. The acid catalyst is typically a mineral acid, e.g., sulfuric acid, but organic acids or reagents like sulfur dioxide can also be used. The low pH drives the hydrolysis of hemicelluloses until near completion resulting in a massive release of sugar. The acid catalysis can also lead to sugar degradation, especially if the pretreatment is long and the temperature high (Fengel and Wegener, 1989). Degradation reactions result in formation of furans, such as furfural and 5-hydroxymethylfurfural (HMF), and carboxylic acids, such as formic acid and levulinic acid, which represent a loss in sugar yield and which are also inhibitory to fermenting microorganisms (Jönsson and Martín, 2016).

Some mild alkaline treatments, mostly with strong bases, are performed in water-biomass systems under temperatures within the typical HTP range. They represent an HTP variation that is sometimes referred to as alkaline hydrothermal pretreatment (Zakaria et al., 2014). In alkaline hydrothermal pretreatment, hydroxide anions resulting from dissociation of the base effectively attack linkages between hemicelluloses and lignin, and they can also promote some cellulose peeling reactions (Kim et al., 2016). The removal of lignin and hemicelluloses results in enhanced enzymatic digestibility of cellulose. Treatments with lime or ammonia are performed at lower temperature and lie outside the HTP classification used here.

Hydrothermal pretreatment with steam explosion (HTP-SE), commonly known as steam explosion, is an extensively investigated method that is used for different raw materials (Galbe and Wallberg, 2019). It can be performed either as an auto-catalytic process or assisted by acidic or alkaline catalysts. HTP-SE can be operated in both batch and continuous modes. Batch mode consists in placing the required amount of biomass in a reactor chamber, and treating it with saturated steam. After a certain time period, from seconds to a few minutes, the reactor is depressurized, and its content is shot into a cyclone, where the pretreated slurry is separated from the stream of steam and volatiles. In continuous systems, which are of special interest for industrial operations, moisturized biomass is screw-conveyed to a plug feeder and forced into the reactor, where steam is applied. Ensuring a controlled time at the work temperature, the material is ejected into a flash tank, where the vent stream flows upwards to a condenser, and the slurry is collected from the bottom (Wang et al., 2015).

HTP-SE is a texturing-hydrolysis process disrupting biomass structure and increasing its porosity. It also causes partial hydrolysis of hemicelluloses, and some fragmentation of cellulose and lignin. Those effects lead to improved susceptibility of cellulose to enzymatic saccharification. There has been controversy on the role of the explosion (Galbe and Wallberg, 2019), which was initially believed to be the main cause of the changes during pretreatment. It has recently been shown that the driving force is the synergy of different actions, including the explosion itself, but also other physical events and chemical reactions (Muzamal et al., 2015). Recent studies comparing steam pretreatment of softwood with and without explosion bring back the attention to the importance of the explosion for enhancing enzymatic digestibility of cellulose (Pielhop et al., 2016).

### Conditions for Hydrothermal Pretreatment

In HTP, biomass and water is heated to from around 150°C to around 220°C for a period ranging from a few minutes to around 1 hour (Ruiz et al., 2020). Pressure of up to around 25 bar is applied, which forces the water to remain in liquid state. Time and temperature are set so that the severity is suitable for achieving effective solubilization of hemicelluloses, activation of cellulose, and minimal formation of by-products. Based on detailed studies on the kinetics of lignocellulosics fractionation by steam-aqueous pretreatments (Overend and Chornet, 1987), a severity factor (SF) has been established. The SF is calculated as the logarithm of the reaction ordinate R_0_:

$$R_{0}=t\;\times \exp\;\left(\frac{T_{i}-100}{14.75}\right)$$ (1)

where *t* is time in minutes, and *T*_*i*_ is temperature in °C (Eq. 1). SF calculations are often restricted to the isothermal period, and exclude the heating and the cooling period. It is more rational to calculate the SF based on temperature profiles built by measurements along the whole process using the equation:

$$R_{0}=\overset{t}{\underset{0}{\int }}\exp\;\left(\frac{T_{i}-100}{14.75}\right)dt\text{, }\mathrm{or}\;SF=log\overset{n}{\underset{i=1}{\sum }}\left[\text{t }\times \exp\;\left(\frac{T_{i}-100}{14.75}\right)\right]$$ (2)

The required severity depends on the type of raw material. For example, lower severity is typically needed for gramineous biomass than for wood. A-HTP at SF 3.8 resulted in good enzymatic convertibility for sugarcane bagasse (Ilanidis et al., 2021b) and wheat straw (Ilanidis et al., 2021c). An SF below 3.0 was not effective, and a value above 4.3 resulted in excessive sugar degradation. A similar trend was reported for sugarcane straw, where SF 3.2 was not enough and 4.1 was optimal (Batista et al., 2019). For poplar, an SF between 4.2 and 4.6 resulted in substantial solubilization of hemicelluloses and positively affected other factors associated with reduced recalcitrance (Li et al., 2017).

In DA-HTP, the combined severity factor (CSF = SF − pH) is an important indicator, but the determination of the pH value is a complication. A common approach is to measure the pH of the liquid after pretreatment (Kellock et al., 2019). Other options are to measure it in the beginning, which might be physically difficult if the solids loading is high, or to calculate it based on the definition of pH.

Other key operational conditions are the solids loading and the amount of added acid or alkali. Solids loading is commonly reported as weight percentage of dry matter in the suspension or as liquid-to-solid ratio (LSR). The percentage approach is rather clear for comparison of reported results, and typical values for A-HTP are ∼5–20% (w/w) depending on the experimental setup. Although the LSR procedure is accurate with regard to inclusion of biomass moisture as part of the liquid fraction, it is often ambiguous considering that the liquid fraction can be given in either weight or volume units.

It is often difficult to compare dosages of acid or alkali in descriptions of DA-HTP or mild alkaline pretreatment, respectively. Dosages can be given in different ways, e.g., as concentration in solutions added to reaction mixtures, as weight percentage based on the whole reaction mixture, or as weight percentage based on dry weight of biomass. For example, a dosage of “1% H_2_SO_4_” might refer to 1) 1% H_2_SO_4_ solution used for preparing a reaction mixture, 2) 1 g H_2_SO_4_ per 100 g suspension, or 3) 1 g H_2_SO_4_ per 100 g dry biomass. Explicit descriptions of the used procedures are required for facilitating interpretation of research efforts in the area.

In some HTP methods, oxygen or air are used for stimulating delignification through wet oxidation. Typically, those oxidative pretreatments are applied to herbaceous biomass, and are assisted by alkaline additives, such as sodium carbonate (Martín et al., 2007) or ammonium hydroxide (An et al., 2019). In a recent study, an oxidative approach was applied to softwood and sugarcane bagasse under acidic conditions, and it was shown that oxygen addition can modulate the severity of hydrothermal pretreatment also under acidic conditions (Ilanidis et al., 2021a).

### Enzymatic Saccharification

Enzymatic saccharification aims at breaking down cellulose and potential residual hemicelluloses into monomeric sugars. It is a synergistic multi-step process that is carried out with heterogeneous enzyme cocktails containing both cellulose-active glycoside hydrolases (GHs) and accessory enzymes (Van Dyk and Pletschke, 2012). These enzymes can be derived from lignocellulolytic fungi or bacteria. However, the most extensively studied cellulase preparations are derived from *Trichoderma reesei* (Kubicek, 2013).

**FIGURE 1:**
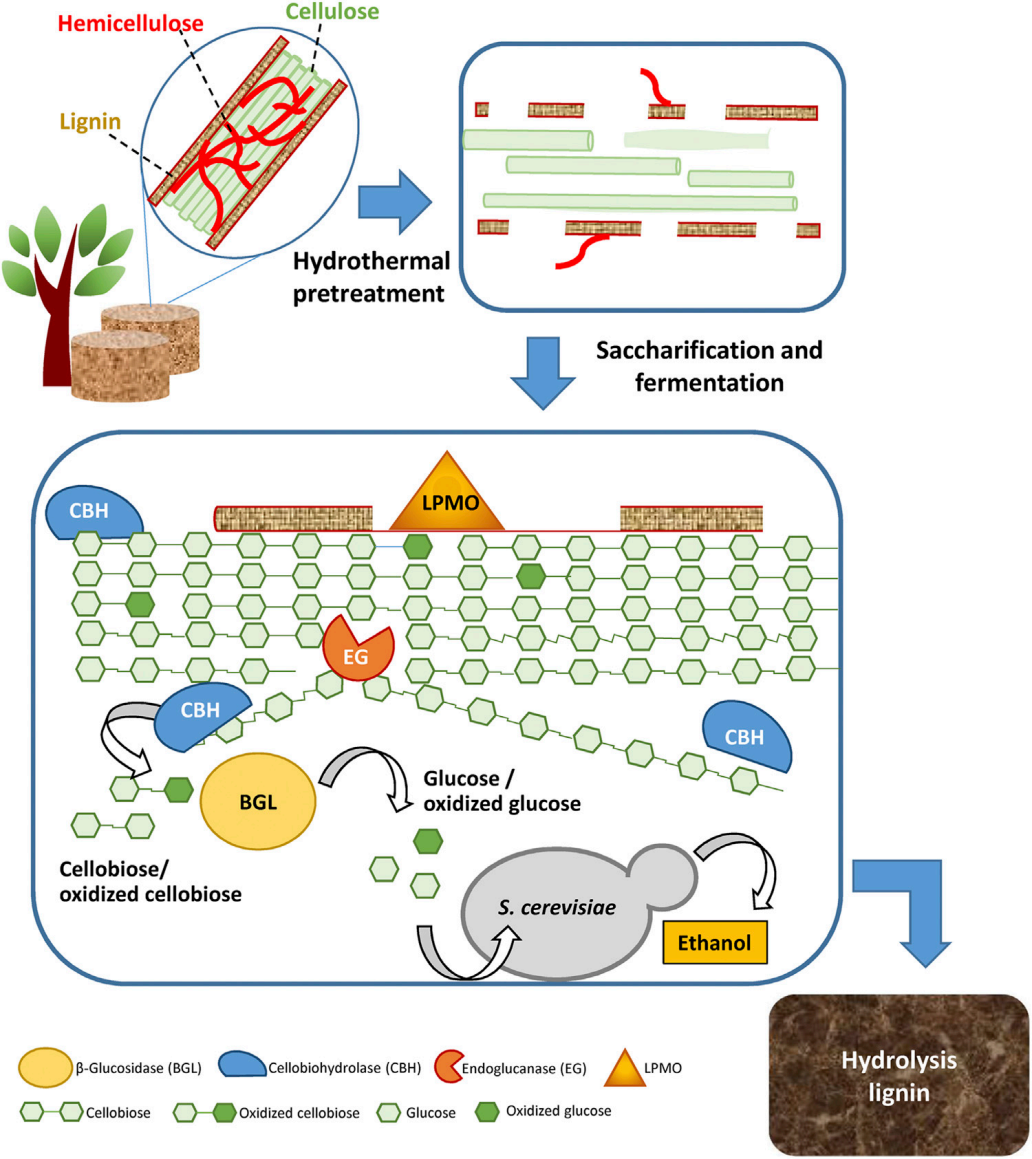
Schematic view of pretreatment, enzymatic saccharification, and fermentation of sugars from lignocellulosic biomass.

Cellulose depolymerization is a complex process, in which enzymes adsorb on the surface of cellulose, get access to the cellulose chains, and catalyze deconstruction of cellulose to glucose (Figure 1). The first two steps are related to cellulose accessibility and amorphogenesis (Arantes and Saddler, 2010; Arantes and Saddler, 2011). Hydrolysis of cellulose to glucose is catalyzed mainly by three enzymes: endoglucanase (EG), cellobiohydrolase (CBH) (or exoglucanase), and β-glucosidase (BGL) (Van Dyk and Pletschke, 2012). EG catalyzes the hydrolysis of interior glycosidic bonds of cellulose chains to oligomeric cellulose chains. CBH attacks the ends of cellulose chains, either at reducing ends (as CBH I) or at the non-reducing ends (as CBH II), thereby incrementally shortening the chains by splitting off the disaccharide cellobiose. BGL catalyzes the hydrolysis of cellobiose to glucose (Figure 1).

Cellulase cocktails are often supplemented with accessory enzymes, such as lytic polysaccharide mono-oxygenase (LPMO) belonging to Auxiliary Activity Family 9 (AA9), formerly GH61. LPMO, which is an oxidoreductase rather than a hydrolase, splits cellulose chains by catalyzing formation of oxidative nicks (Figure 1). By creating shorter glucan chains, LPMO acts synergistically with hydrolases and accelerates the saccharification of lignocellulosic biomass (Horn et al., 2012). Thus, the catalytic action of LPMO is covered by the term saccharification, whereas the term hydrolysis covers most, but not all, reactions occurring during enzymatic saccharification. Other important accessory enzymes include various hemicellulases, such as xylanase, xylosidase, mannanase, mannosidase, α-glucuronidase, α-arabinosidase, acetyl xylan esterase, and others. They act in concert to remove residual hemicelluloses and thereby further improve cellulose accessibility in pretreated biomass (Hu et al., 2011).

Slurries recovered after hydrothermal pretreatment have acidic pH (often around pH 2), and hence the pH needs to be adjusted (typically to around 5). Incubation of reaction mixtures is typically performed with agitation at 45–50 °C for 24–72 h. Enzyme dosages are based on enzyme activity units, enzyme protein, volume or mass fractions of enzyme preparations (in which enzyme protein may account for only a minor fraction), or even molarity. Common examples include filter paper units (FPU), carboxymethyl cellulose units (CMCase units), enzyme protein per dry weight of biomass or glucan, and enzyme preparation per dry weight of biomass or glucan.

Preparative enzymatic saccharification typically aims at obtaining as high sugar yield as possible in an economically sound way. In contrast, analytical enzymatic saccharification aims at comparison of the susceptibility of different biomass preparations to enzymatic saccharification, comparison of different pretreatment methods or other experimental conditions, or comparison of different enzyme preparations (Gandla et al., 2018). Therefore, analytical enzymatic saccharification is typically not exhaustive, as that would blur the result of the comparisons.

In an industrial context it would be desirable to have a high product titers, and, therefore, enzymatic saccharification of lignocellulosic biomass at high solids loading (such as a content of water-insoluble solids above 15%) would be preferable (Kristensen et al., 2009). However, the use of high solids loadings is often associated with operational challenges and sugar yields are typically comparatively low.

### Microbial Fermentation

‘Fermentation’ can be used in a broad and a narrow sense. In a broad sense, ‘fermentation’ unspecifically refers to a cultivation of a microorganism. In a narrow sense, it is an anaerobic energy-yielding metabolic activity in which organic substances serve as both electron donor and electron acceptor. Typical electron acceptors are pyruvate or a derivative of pyruvate, and common products include lactic acid and ethanol.

Sugars derived from pretreatment and enzymatic saccharification of lignocellulosic biomass are typically fermented to cellulosic ethanol using yeast (such as *Saccharomyces cerevisiae*) or bacteria (such as *Zymomonas mobilis*). As 2 mol of ethanol (46.1 g/mol) can be obtained from 1 mol of glucose (180 g/mol) (Eq. 3), the maximum theoretical yield is (2 × 46.1)/180 = 0.51 g ethanol per g glucose.

$${\text{C}}_{6}{\text{H}}_{12}{\text{O}}_{6}{\;=\;2\text{ CH}}_{3}{\text{CH}}_{2}{\text{OH }+\;2\text{ CO}}_{2}$$ (3)

As cellulose consists of glucose units (on average 162 g/mol) and as a consequence of water (18 g/mol) addition to glycosidic linkages during hydrolysis of cellulose to glucose, the maximum theoretical yield from 1.00 g cellulose is 180/162 = 1.11 g glucose. Thus, the maximum theoretical yield from 1.00 g cellulose is 1.11 × 0.51 = 0.57 g ethanol. Common alternative fermentation products include biobutanol and lactic acid, which can be produced via ABE (acetone-butanol-ethanol) and LAB (lactic acid bacteria) fermentation, respectively (Sivanarutselvi et al., 2017; Tu et al., 2019).

Saccharification and fermentation can be carried out using various configurations, such as Separate Hydrolysis and Fermentation (SHF) (Galbe and Zacchi, 2002), Simultaneous Saccharification and Fermentation (SSF) (Galbe and Zacchi, 2002), Hybrid Hydrolysis and Fermentation (HHF) (Teter et al., 2014), and Consolidated Bio-Processing (CBP) (Lynd et al., 2017). SHF is a two-step process where enzymatic saccharification of pretreated biomass and microbial fermentation of sugars are carried out sequentially. Advantages include that optimal conditions can be used for both enzymatic saccharification and microbial fermentation, and that the residual solids, the hydrolysis lignin, can be separated from the liquid phase, the hydrolysate, before microbes are added. However, sugars will accumulate during the process and cause end-product inhibition of cellulolytic enzymes. In SSF, enzymatic saccharification and microbial fermentation are carried out in parallel as a one-step process. Since optimal temperatures for enzymatic saccharification (typically around 50°C) and fermentation (typically around 30°C) differ, and since optimal pH and aeration conditions may also differ, the conditions used for SSF are a compromise and are thus suboptimal for at least some of the biocatalysts involved. At least to some extent, end-product inhibition by sugars is avoided, but it is more challenging to recycle the microorganism as it will be mixed with hydrolysis lignin. HHF is a two-step process, in which the first step only includes enzymatic saccharification and the second step includes both enzymatic saccharification and microbial fermentation. In the first step, enzymatic saccharification can be carried out under optimal conditions, but the second step suffers from the same drawbacks as SSF. In CBP, production of enzymes, saccharification of pretreated biomass, and fermentation of sugars are carried out as a one-pot process. Thermophilic anaerobic bacteria, such as *Clostridium thermocellum* (Olson et al., 2015), and *S. cerevisiae* engineered to produce cellulases (Kroukamp et al., 2018) are two options that have been considered.

Microbial fermentation processes can also be carried out in batch, fed-batch, and continuous mode. In batch mode, all medium is added directly to the reactor in the beginning of the process and the final culture volume is similar to the initial volume. In fed-batch mode, the fermentation is initiated using a minor portion of the medium, and then more medium is gradually fed into the bioreactor until it is full or all medium has been consumed. In a continuous fermentation process, the medium is fed into the bioreactor reactor continuously, but is also continuously removed. In Brazil, 83% of distilleries that produce first generation bioethanol rely on fed-batch processes, while continuous processes account for only 17% (Godoy et al., 2008).

### Factors Affecting Biomass Recalcitrance

The resistance to biochemical processing of lignocellulosic feedstocks is known as “recalcitrance”. Recalcitrance refers to barriers to access of enzymatic and microbial biocatalysts to carbohydrates in the biomass. There are multiple interrelated factors that contribute to recalcitrance (Table 3), and they include both factors related to the physical structure of the biomass and factors related to the chemical composition (Wang et al., 2018a). Structural features that affect biomass recalcitrance include the highly organized architecture of secondary cell walls, the particle size, the porosity, and the accessible surface area of cellulose. Chemical features include hemicelluloses and lignin, substances that create physical barriers that limit the accessibility of enzymes to cellulose (Zhao et al., 2012b). Higher fractions of hemicelluloses and lignin in the material obviously contribute to recalcitrance, but some investigations indicate that also certain chemical features of hemicelluloses and lignin affect the recalcitrance. Examples of that include the prevalence of different types of building blocks (i.e., the chemical composition of hemicelluloses and lignin), and the acetylation of hemicelluloses (Chen et al., 2012; Herbaut et al., 2018). The DP and the crystallinity of the cellulose may also affect recalcitrance (Kumar et al., 2009).

**TABLE 3:** Factors affecting recalcitrance of lignocellulosic feedstocks, and common detection methods with references^a^.

Recalcitrance factor	Common detection methods	References
Cellulose accessibility	Simons’ staining	Chandra et al. (2008)
Cellulose crystallinity	XRD	Kumar et al. (2009)
Cellulose DP	GPC	Kumar et al. (2009)
Cell wall architecture	Fluorescence microscopy, SEM	Wang et al. (2018a)
Hemicellulose acetylation	HPAEC, NMR, OLIMP	Wang et al. (2020)
Hemicellulose and lignin content	Analytical acid hydrolysis combined with HPLC or HPAEC	Ilanidis et al. (2021b)
		Ilanidis et al. (2021d)
Particle size	Sieving	Wang et al. (2018b)
Porosity	BET analysis	Wang et al. (2020)
S:G ratio	Py-GC/MS	Ilanidis et al. (2021b)
		Ilanidis et al. (2021d)

a BET, Brunauer–Emmett–Teller; DP, degree of polymerization; FTIR, Fourier-transform infrared spectroscopy; GC, gas chromatography; GPC, gel permeation chromatography; HPLC, High-pressure liquid chromatography; NMR, nuclear magnetic resonance; OLIMP, oligosaccharide mass profiling, Py-GC/MS, Pyrolysis-gas chromatography/mass spectrometry; SEM, scanning electron microscopy; S:G ratio, ratio of syringyl units and guaiacyl units in lignin; XRD, X-ray diffraction.

Hydrothermal pretreatment removes hemicelluloses, modifies cellulose and lignin, creates cell-wall disorder, and increases biomass porosity and cellulose accessibility (Wang et al., 2018a). It is noteworthy that hydrothermal pretreatment under acidic conditions increases the fraction of lignin and the cellulose crystallinity, but still improves biomass digestibility (Foston and Ragauskas, 2010). Although an increased fraction of lignin and an increase in cellulose crystallinity should theoretically increase the recalcitrance, the positive effects of the pretreatment, such as disruption of cell wall architecture and a decreased fraction of hemicelluloses, are evidently much more important for recalcitrance, and overshadow the negative effects and lead to greatly improved enzymatic digestibility. In line with that, a recent study on *Eucalyptus* wood indicated that, on the one hand, pretreatment effects such as partial removal of lignin, increased S/G ratio in lignin, and lowered cellulose crystallinity exhibited no significant positive effects (Thoresen et al., 2021). On the other hand, effects such as disruption of cell wall architecture, exposure of fibres and increased cellulose accessibility, and substantial removal of hemicelluloses had a positive impact on enzymatic digestibility. Evidently, the impact of different recalcitrance factors varies greatly. Consequently, the impact of recalcitrance factors of relatively low importance, such as cellulose crystallinity, may not be easily observed in complex systems, where recalcitrance factors of high importance, such as preserved cell wall architecture and high hemicellulose content, are predominant.

## By-Products of Hydrothermal Pretreatment

### Pretreatment Liquid

After hydrothermal pretreatment, typically under acidic conditions, the liquid phase will contain organic substances such as aliphatic aldehydes, aliphatic carboxylic acids, benzoquinones, disaccharides, furans (such as furan aldehydes and furoic acids), monosaccharides, oligosaccharides, phenylic substances (phenolic as well as non-phenolic aromatics), and uronic acids (Jönsson and Martín, 2016). These are summarized in Table 4, which also indicates their main precursor(s) and contains examples of references that address occurrence, formation, and analysis. Whereas glucose is a product that fermenting microorganisms can utilize under anaerobic or oxygen-limited conditions, this is not always the case for other lignocellulose-derived monosaccharides, disaccharides, and oligosaccharides. Disaccharides and oligosaccharides can tentatively be converted to monosaccharides using post-hydrolysis (Shevchenko et al., 2000), an approach that is also used for analytical purposes, or by using enzyme cocktails that not only degrade cellulose but also assist degradation of dimeric and oligomeric saccharides and make them available to microorganisms. Inhibitory effects of by-products and conditioning of lignocellulosic hydrolysates was recently reviewed (Jönsson and Martín, 2016), and this subsection will therefore be restricted to recently discovered inhibitors and novel findings about enzyme inhibition.

**TABLE 4:** Groups of products and by-products solubilized by hydrothermal pretreatment.

Group	Description/Examples	Main precursors	References
Aliphatic aldehydes	Acetaldehyde, formaldehyde	Probably lignin (formaldehyde) and hemicelluloses (acetaldehyde)	Cavka et al. (2015)
Aliphatic carboxylic acids	Acetic acid, formic acid, levulinic acid	Hemicelluloses, cellulose; acetic acid from acetyl groups; sugar degradation	Du et al. (2010)
Benzoquinones	*p*-Benzoquinone, 2,6-dimethoxybenzoquinone	Lignin, phenolic extractives	Stagge et al. (2015)
Disaccharides	Cellobiose, xylobiose	Hemicelluloses, cellulose	Xu et al. (2013)
Furans	Heteroaromatics such as furfural, HMF, 2-furoic acid	Hemicelluloses, cellulose; sugar degradation	Du et al. (2010)
Monosaccharides	Arabinose, galactose, glucose, mannose, xylose	Hemicelluloses, cellulose	Shevchenko et al. (2000)
Oligosaccharides	Glucooligosaccharides, xylooligosaccharides	Hemicelluloses, cellulose	Xu et al. (2013)
Phenylic compounds	Phenolic and non-phenolic aromatic compounds	Lignin, phenolic extractives	Du et al. (2010)
Uronic acids	Galacturonic acid, glucuronic acid, 4–*O*-methyl-glucuronic acid	Hemicelluloses	Wang et al. (2018a)

Benzoquinones, such as *p*-benzoquinone and 2,6-dimethoxybenzoquinone, were found to be ubiquitous in pretreated biomass, albeit in very low concentrations (for *p*-benzoquinone up to around 6 mg/L or 60 μM) (Stagge et al., 2015). However, as *p*-benzoquinone exhibited an inhibitory effect on *S. cerevisiae* already at around 20 μM, its high molecular toxicity nevertheless makes it relevant as an inhibitor. Furthermore, benzoquinones are oxidation products of benzenediols, such as hydroquinone. The presence of oxidants and reductants, and the handling and storage of pretreated biomass may therefore affect formation and occurrence (Martín et al., 2018).

Formaldehyde and acetaldehyde were found to be prevalent in pretreated biomass in concentrations up to ∼4 and ∼2 mM, respectively (Cavka et al., 2015). Although lignin is probably the main precursor for formaldehyde, other lignocellulosic constituents, including extractives and polysaccharides, could be other sources. Acetyl groups in hemicelluloses are a tentative precursor for acetaldehyde, although this has to be investigated in more detail. Unsurprisingly considering its central role in the metabolic pathway of ethanolic fermentation, acetaldehyde was much less toxic to yeast than formaldehyde (Cavka et al., 2015). Formaldehyde was found to be the single most important inhibitor of yeast in hydrothermally pretreated softwood (Martín et al., 2018). Although phenylic inhibitors and synergistic effects of different groups of inhibitors also play a role, the discovery of previously unknown inhibitors, such as formaldehyde and *p*-benzoquinone, can help explaining toxic effects of lignocellulosic hydrolysates containing non-toxic levels of furan aldehydes and aliphatic acids.

Inhibition of cellulose-degrading enzymes by pretreatment by-products differs from inhibition of microorganisms. Monomeric sugars causing end-product inhibition of cellulolytic enzymes and phenolic substances have been found to be major contributors to enzyme inhibition in the liquid fraction of steam-pretreated biomass (Zhai et al., 2016). Oligosaccharides produced during pretreatment could also contribute to enzyme inhibition (Kumar and Wyman, 2014). There are several direct studies of the inhibitory effects of phenols on enzymes (e.g., Ximenes et al., 2011; Zhai et al., 2018). Phenols may exert several effects on enzymes. Conditioning of lignocellulosic hydrolysates using sulfur oxyanions, such as sodium sulfite or sodium dithionite, alleviated inhibition of both yeast and enzymes, whereas conditioning using sodium borohydride only alleviated inhibition of yeast (Cavka and Jönsson, 2013). A difference between sulfur oxyanions, on the one hand, and sodium borohydride, on the other hand, is the capacity of the former to strongly hydrophilize inhibitors by sulfonation (Cavka and Jönsson, 2013; Jönsson and Martín, 2016). This suggests that hydrophobic interactions is one cause of inhibition of enzymes, whereas inhibition of microorganisms is more related to reactivity and interference of metabolism. In line with that, studies of steam-pretreated woody biomass have indicated that hydrophobic phenolics are the major inhibitory compounds for enzymes (Zhai et al., 2018).

### Solid Phase

Pseudo-lignin is an aromatic Klason-lignin-positive substance derived from carbohydrates during thermal treatment of biomass, including hydrothermal pretreatment (Shinde et al., 2018). Pseudo-lignin is typically derived mostly from hemicelluloses, such as xylan, which are more heat labile than cellulose and decompose to form pseudo-lignin at lower temperatures (Normark et al., 2016). In contrast, real lignin is a polymeric substance consisting of phenylpropane units, and it is formed by combinatorial cross-linking of radicals created by oxidation of monolignols (Ralph et al., 2019). The monolignols are formed via the shikimate pathway, which is also involved in the biosynthesis of aromatic amino acids. Thus, although pseudo-lignin and real lignin share some common features, such as aromaticity, insolubility in water under neutral conditions, and acid-resistance (i.e., being Klason-lignin-positive substances), their basic chemical structure and their origin are fundamentally different.

Pseudo-lignin formation represents a yield loss, as the fraction of carbohydrates that form pseudo-lignin is not converted to fermentable sugars. Pseudo-lignin also has a negative impact on enzymatic saccharification of cellulose. This negative impact is caused by reduced cellulose accessibility and by catalytically non-productive binding of enzymes to pseudo-lignin (Kumar et al., 2013; Wang and Jönsson, 2018).

### Gas Phase

Some volatile substances originating from the biomass and from the pretreatment reactions have been captured from pretreatment vapor and analyzed using mass spectrometry. Condensate from steam-explosion of corn stalks contained furans, phenols, and carboxylic acids (Yang et al., 2017). Main constituents, as judged from peak areas, were furfural, phenol, and 4-hydroxy-butanoic acid. Phenol was the predominant analyte in non-condensable gas collected using dichloromethane (Yang et al., 2017). Analysis of a set of condensates from pretreatment of sugarcane bagasse showed that furfural accumulated in the condensates and that the levels were related to sugar degradation and furan aldehyde formation during pretreatment (Ilanidis et al., 2021b). Further investigations are needed to characterize volatile substances formed during hydrothermal pretreatment of biomass and to understand their potential contribution to the mass balance of hydrothermal pretreatment.

## Characterization of Pretreated Lignocellulose

Pretreatment of lignocellulosic materials results in a slurry of variable consistency depending on the pretreatment method and the initial solids loading. Based on the downstream strategy, the slurry can either be separated into a solid fraction (i.e., pretreated solids) and a liquid fraction (i.e., pretreatment liquid or hemicellulose hydrolysate) to be processed separately, or be used directly as it is for conditioning and biocatalytic conversion. Regardless of the processing strategy, a thorough characterization of the pretreated material is required in order to assess the efficiency of the pretreatment. That includes determination of the gravimetric yield of solids, the chemical composition of the solid and liquid phases, evaluation of the recovery of the main organic components of biomass, and assessment of enzymatic digestibility and fermentability. An overview of methodology useful for characterization of pretreated biomass is provided below.

### Slurry Characteristics

The characteristics of slurries produced during the pretreatment step have a critical effect on subsequent processing steps. The dry-matter (DM) content of a slurry is comprised of insoluble solids, often referred to as water-insoluble solids (WIS), and soluble solids (SS). Main components of SS include organic degradation products derived from hemicelluloses (such as sugars), some degradation products from cellulose and lignin, hydrophilic extractives, and salts. WIS is composed mainly of cellulose and lignin, and typically some residual hemicelluloses.

The DM of the slurry can be measured by using an oven or an automatic infrared moisture analyzer. In both cases, a certain amount of slurry, usually 2–5 g, is heated at 105 °C until a constant mass is reached (Sluiter et al., 2008a). To determine the fractions of WIS and SS, the first step is separation of the solid and the liquid phase (Sluiter et al., 2008b). Vacuum filtration is commonly used for separation. However, if clogging of filters is a problem, centrifugation may serve as an alternative. To determine the SS content, the liquid fraction is passed through a 0.2 µm filter. After that, about 10 mL of the filtrate is dried, typically using an oven, and the residual solids are analyzed gravimetrically. To determine the WIS content, the solid fraction obtained after filtration or centrifugation could be washed several times with deionized water to remove residual liquor. The wash is finished when the glucose concentration in the wash water is < 0.05 g/L. The washed solids are then dried at 105°C using one of the previously mentioned methods. Alternatively, the determination can also be performed without washing (Weiss et al., 2010).

### Analysis of Liquid Phase

Hydrothermal pretreatment fractionates biomass by solubilizing hemicelluloses into monomeric, dimeric, or oligomeric products in the liquid fraction of a biomass slurry, while keeping cellulose and lignin relatively intact in the solid fraction (Galbe and Wallberg, 2019). Characterization of pretreatment liquids to determine the content of sugars as well as by-products helps in evaluating hydrothermal pretreatments aiming at maximal conversion of polysaccharides into sugars. Mild hydrothermal pretreatment would typically result in the formation of substantial amounts of disaccharides and oligosaccharides. Hence, a mild post-hydrolysis of the pretreatment liquid can be done by sulfuric acid at a concentration of 4% (w/w) at 121°C for 60 min, to completely hydrolyze dimeric and oligomeric saccharides to monomers. Monosaccharides generated after hydrothermal pretreatment and post-processing of liquids, such as arabinose, galactose, glucose, mannose, xylose, are generally detected either through high-performance liquid chromatography (HPLC) combined with refractive-index detection (RID) or through high-performance anion-exchange chromatography (HPAEC) combined with pulsed-amperometric detection (PAD). The latter approach typically offers superior resolution and higher sensitivity (Gandla et al., 2018).

Heteroaromatic degradation by-products, such as furfural and HMF, are typically determined using HPLC with UV detection or DAD (diode-array detection). Aliphatic acids, such as acetic acid, levulinic acid, and formic acid, are typically analyzed using HPLC or using HPAEC with conductivity detection (Du et al., 2010; Gandla et al., 2018). Splitting of β-O-4 ether bonds of lignin during hydrothermal pretreatment generates phenylic substances, many of which are phenolic. Mononuclear aromatic substances are typically detected using mass spectrometry (Du et al., 2010; Martín et al., 2018).

Apart from determination of individual substances, group analyses are also useful. Determination of total phenolics in pretreatment liquid is often carried out using the Folin-Ciocalteu colorimetric assay with vanillin as a calibration standard (Persson et al., 2002). The total aromatic content (TAC) of pretreatment liquids is analyzed using UV absorbance at 280 nm (Wang et al., 2018b). TAC analysis covers both heteroaromatics, such as furans, and aromatics, such as phenolic and non-phenolic substances. The total carboxylic acid content (TCAC), which covers both aliphatic and aromatic carboxylic acids, is determined by titration (Wang et al., 2018b).

### Analysis of Solid Phase

Determination of the composition of biomass by hydrolysis of the polysaccharides using sulfuric acid has been used for more than a hundred years. The method was further developed by the introduction of two-step treatment with sulfuric acid (TSSA), in which the second step is performed with more diluted acid but at higher temperature. Today, NREL/TP-510–42618 (Sluiter et al., 2012) is a commonly used protocol for performing TSSA. The biomass needs to be extracted prior to TSSA. The solvent could be, for example, ethanol, acetone, a mixture of petroleum ether and acetone, or a mixture of cyclohexane and acetone. A less polar solvent or mix of solvents is useful for efficient extraction of resin from softwood. Biomass extractives are typically measured gravimetrically after solvent evaporation, whereas individual extractives are analyzed using mass spectrometry.

The dried solid residue obtained after extraction is fractionated using 72% (w/w) sulfuric acid for 1 h at 30°C. The mixture is then diluted to 4% (w/w) sulfuric acid with deionized water and is autoclaved at 121°C for 1 hour. Vacuum filtration is applied for separation. The solid fraction is composed of Klason lignin (acid-insoluble lignin) and ash, while sugars and acid-soluble lignin (ASL) are found in the liquid fraction. The monosaccharides are often analyzed using HPAEC-PAD, which offers better resolution and higher sensitivity than HPLC-RID. Klason lignin is determined gravimetrically and ASL is estimated using UV/Vis spectrophotometry. The recommended wavelength and absorptivity used for ASL determination vary depending on the type of biomass investigated (Sluiter et al., 2012). Wavelengths used for ASL determination are in no way specific for lignin, but are subject to interference, e.g., by the presence of furans. The conditions used in TSSA for hydrolysis of the carbohydrates are important for the accuracy of the method. Too low severity will result in residual cellulose in the solid fraction leading to underestimation of the carbohydrate content and overestimation of the Klason lignin content. Too high severity will result in degradation of sugars to furans and carboxylic acids leading to underestimation of the carbohydrate content and, if sugar degradation products are not analyzed and accounted for, poor mass balance (Wang et al., 2017). Although the hydrolysis conditions mentioned above are suitable in many cases, they are not optimal for all sorts of biomass and processed biomass samples.

Ash or mineral content is typically determined gravimetrically after heating at 550–600°C. Ash content may refer to the total ash in non-extracted biomass or to acid-insoluble ash, i.e., the fraction of ash left in the residue after hydrolysis of carbohydrates using the TSSA method. The ash content varies greatly depending on plant species but also depending on the type of tissue. A review of data from 144 species of lignocellulosic biomass (Tao et al., 2012) indicated that the ash content of dry biomass varied between 0.1 and 26.2% (with a mean of 3.5%). The ash content of woody biomass (0.1–6.4% with a mean of 1.9%) was typically lower than that of non-woody biomass (1.0–26.2%, with a mean of 7.0%) (Tao et al., 2012).

Pyrolysis-gas chromatography/mass spectrometry (Py-GC/MS) is another way to compare the contents of carbohydrates and lignin in biomass samples, although the method provides no detailed information on the carbohydrate composition. Initially, the biomass is pyrolyzed at around 500°C in the presence of a gas stream consisting of helium, nitrogen, or argon gas. The fragments are separated by GC and identified by mass spectrometry (Gerber et al., 2012). The method is useful for determination of the ratio of lignin subunits, i.e., the S:G or S:G:H ratio.

Analysis using solution-state and solid-state nuclear magnetic resonance (NMR) is useful for obtaining information about the effects of pretreatment on biomass (Lu and Ralph, 2011; Bryant et al., 2020). Solid-state NMR offers relatively low resolution and sensitivity, but has the advantage that the original structure of the biomass sample is maintained. Methods such as solid-state cross-polarization/magic angle spinning (CP/MAS) ^13^C NMR are therefore useful for studies of the crystallinity and ultrastructure of cellulose. Solution-state NMR offers higher resolution and sensitivity, and can provide detailed information on lignin and hemicelluloses. Two dimensional heteronuclear single quantum coherence (2D HSQC) NMR offers detailed information on the composition and linkages of lignin and biomass polysaccharides. However, dissolution typically causes some degradation of the sample, and information on cellulose crystallinity is lost.

Infrared light is applied in Fourier-transform infrared (FTIR) spectroscopy to scan lignocellulosic materials and study structural features before and after pretreatment. FTIR is a simple and fast analytical technique with easy sample preparation that identifies functional groups in a semi-quantitative manner. It has sometimes been used to estimate changes in CrI (crystallinity index). Absorption bands between 800 and 1800 cm^−1^ are assigned to the main components of lignocellulosic materials (Faix and Böttcher, 1992).

X-ray diffraction (XRD) is commonly used to estimate the crystallinity of cellulose in biomass. However, CrI values might be affected both by the methodology and by factors such as moisture content (Agarwal et al., 2017).

Pseudo-lignin might be difficult to distinguish from real lignin as both substances end up as Klason lignin in compositional analysis using TSSA. Several different methods can be used to get an indication of the occurrence of pseudo-lignin (Shinde et al., 2018). An approach to estimate the pseudo-lignin content is to combine compositional analysis using TSSA with Py-GC/MS. The Klason lignin determined using TSSA will include both real lignin and pseudo-lignin, but in analysis using Py-GC/MS pseudo-lignin will be included in the carbohydrate fraction rather than in the lignin fraction. Hence, a relative value for the pseudo-lignin content can be estimated by subtracting the peak area fraction assigned to lignin in the Py-GC/MS analysis from the mass fraction of lignin determined using compositional analysis with TSSA (Normark et al., 2016; Ilanidis et al., 2021a).

The Simons’ staining method gives a relative estimate of cellulose accessibility based on dye adsorption using Direct Blue (DB) and Direct Orange (DO). The larger DO dye (>100 kDa) has affinity for cellulose and a size that is as large or even larger than that of many enzymes. The DB dye, which is smaller than the enzymes, populates only small pores and is less relevant with regard to determination of cellulose accessibility. The difference between final and initial concentrations of each dye indicates the amounts of adsorbed dyes (Chandra et al., 2008).

The Brunauer–Emmett–Teller (BET) method is useful for investigating the specific surface area and the pore-size distribution of biomass before and after pretreatment (Wang et al., 2020). The BET method is typically based on monitoring adsorption of nitrogen gas on the sample surface.

Surface modifications, fragmentation of biomass, and changes in cell wall integrity as a result of pretreatment can be investigated using microscopy, including light microscopy, electron microscopy, atomic force microscopy, and fluorescence microscopy. Staining with dyes such as phloroglucinol-HCl, which causes purple staining of cinnamaldehydes in lignin, is a way to use light microscopy for qualitative assessment of changes in cell wall architecture during pretreatment (Thoresen et al., 2021). Electron microscopy results in two-dimensional images, while atomic force microscopy provides three-dimensional images with high resolution (Karimi and Taherzadeh, 2016). Due to the autofluorescence of lignin (Donaldson, 2020), fluorescence microscopy is useful for studying the effects of pretreatment on cell wall architecture and the distribution of lignin (e.g., Wang et al., 2018a).

Fragmentation of biomass during pretreatment can be analyzed using sieving or image analysis. Using a series of sieves in the range 50–800 μm, pretreated softwood was fractionated into four size ranges (dust, 50–100 μm; fine particles, 100–500 μm; small particles, 500–800 μm; large particles, >800 μm) and quantitated (Wang et al., 2018b). The particle size distribution was found to be correlated (*R* ^2^ ≥ 0.96) to the ASL content of pretreated softwood, the glucose content in the pretreatment liquid, and to the enzymatic digestibility.

### Recoveries

**FIGURE 2:**
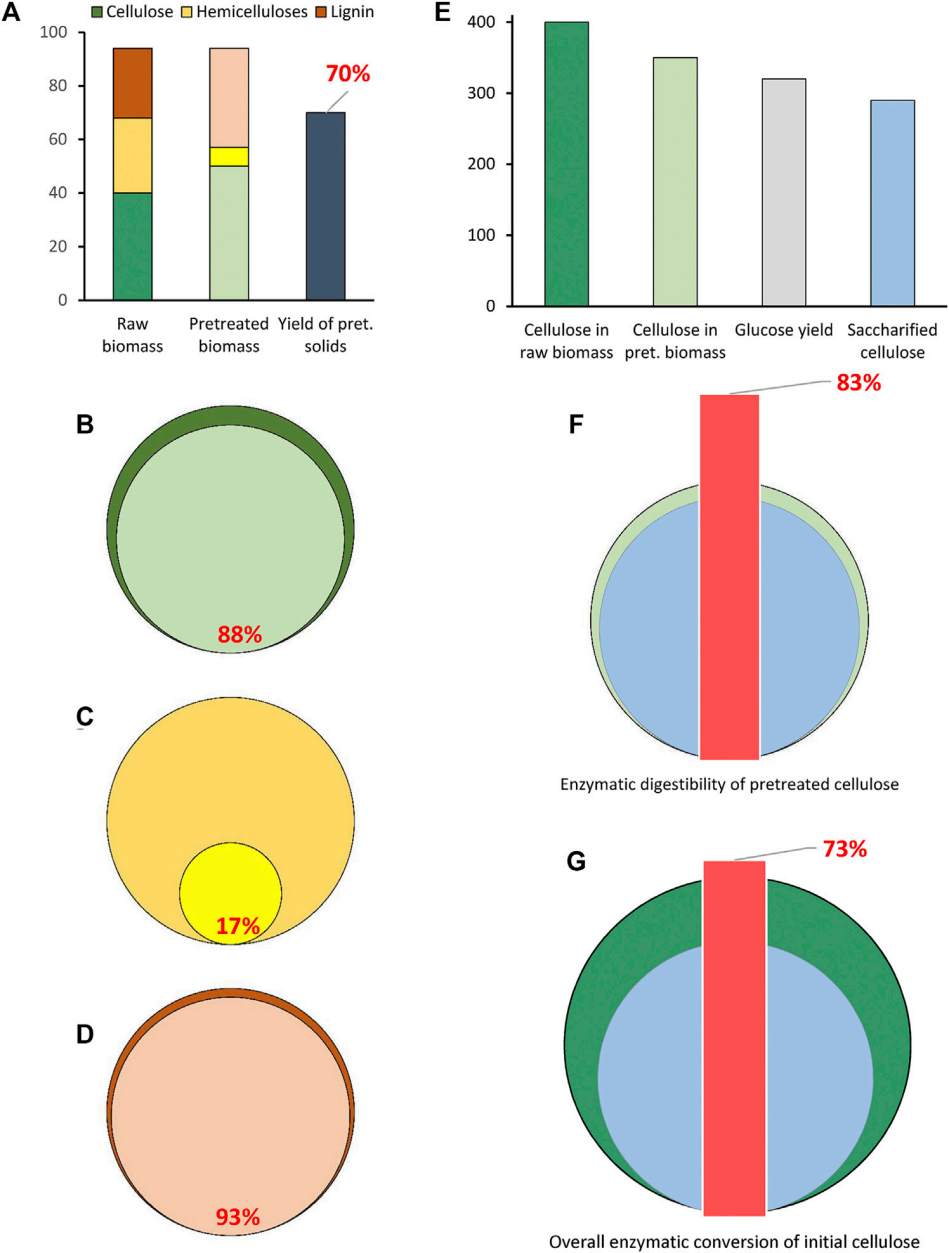
Graphical representation of recovery of the main components in pretreated solids **(A–D)** and enzymatic saccharification indicators **(E–G)**. Input parameters **(A)** for recovery calculations (%) for cellulose **(B)**, hemicelluloses **(C)**, and lignin **(D)** in pretreated solid biomass. Input parameters **(E)** for calculation of enzymatic saccharification indicators (kg/ton initial biomass); enzymatic digestibility of pretreated cellulose (%) **(F)**; overall enzymatic conversion of initial cellulose (%) **(G)**. In **(B)**, **(C)** and **(D)**, internal circles represent the recovery of each component in pretreated biomass and external circles represent the initial amount (100%) of the component in raw biomass. In **(F)** and **(G)**, the internal circles represent saccharified cellulose, and the external ones represent cellulose in pretreated **(F)** or raw **(G)** biomass.

The compositional analysis is not enough for having a full picture of the fate of lignocellulose constituents during pretreatment (Figure 2A). In an example with a hypothetical lignocellulosic material processed through HTP, the composition of raw biomass (Figure 2A, left column) and pretreated solids (Figure 2A, middle column) points at an apparent increase of the contents of cellulose and lignin, from 40 to 50% and from 26 to 38%, respectively, while the content of hemicelluloses drops from 28 to 7%. A better representation is provided by mass balances, which disclose the recoveries of biomass constituents after pretreatment. Considering an input of 100 kg raw biomass with an output of 70 kg of pretreated solids (Figure 2A, right column), the recovered cellulose (Eq. 4) would be:

$$Cellulose\;recovery\;\left(\%\right)=\frac{Cellulose\;in\;pret.\;solids\;\times \;Pret.\;solids\;weight}{Cellulose\;in\;raw\;biomass\times Raw\;biomass\;weight}\times 100\;=\frac{50\;\times \;70}{40\times 100}\times 100=88\%$$ (4)

…which is graphically shown in Figure 2B. This reveals that 12% of initial cellulose was not recovered in the pretreated solids. Non-recovered cellulose corresponds to a minor fraction that is susceptible to hydrolysis during pretreatment, primarily amorphous cellulose, and it ends up in the liquid stream as, for example, glucose or degradation products such as HMF, formic acid, and levulinic acid. The same calculations reveal recoveries of 17% (Figure 2C) and 93% (Figure 2D), respectively, for hemicelluloses and lignin. The actual carbohydrate availability for bioconversion and the amount of sugar lost during pretreatment can be elucidated if the liquid fraction is included in the mass balances. It should be noted that apparent lignin recovery over 100% can be achieved in some experiments. That happens when pseudo-lignin formation occurs in combination with low solubilization of real lignin. This is typical for DA-HTP, but it happens also for A-HTP under severe conditions.

### Enzymatic Digestibility and Enzyme Inhibition

Quantification of changes in the susceptibility of cellulose to enzymatic saccharification after pretreatment is crucial for assessing the efficiency of the applied method. The enzymatic digestibility (or convertibility) based on cellulose contained in the sample subjected to saccharification is often reported. Although that indicator shows how susceptible to saccharification the cellulose that remained in the solids after pretreatment is, it fails to capture the fact that a fraction of the cellulose was lost during pretreatment. A more rational approach is the overall conversion, which is based on the cellulose contained in the raw biomass.

Below, a calculation example based on the parameters given in Figure 2E is provided. One ton of a biomass material containing 400 kg cellulose is subjected to pretreatment. A cellulose recovery of 88%, i.e., 350 kg, results from the pretreatment. An aliquot of pretreated solids suspended in a buffer solution and submitted to enzymatic saccharification results in 320 kg glucose, which corresponds to 290 kg of saccharified cellulose. The amount of glucose is higher than that of cellulose because, due to water addition to glycosidic linkages, the mass of sugars resulting from hydrolysis of hexosans is increased by a factor of 1.11 compared to the polysaccharide mass. The enzymatic digestibility (ED), calculated as indicated in Eq. 5,

$$ED\;\left(\%\right)=\frac{Saccharified\;cellulose}{Cellulose\;submitted\;to\;saccharification}\times 100=\frac{290}{350}\times 100=83\text{\%}$$ (5)

indicates that 83% (w/w) of the cellulose contained in the pretreated solids is susceptible to conversion to glucose under the conditions applied (Figure 2F). The overall conversion (OC), can be calculated as indicated in Eq. 6, and it shows that 73% (w/w) of cellulose contained in the raw material is saccharified upon pretreatment and enzymatic saccharification (Figure 2G).

$$OC\;\left(\%\right)=\frac{Saccharified\;cellulose}{Cellulose\;in\;raw\;biomass}\times 100,\text{ or}\;\text{as}\;\;OC\;\left(\%\right)=\frac{ED\;\times \;Cell.\;\;recovery\;in\;pret.}{100}\;=\;\frac{83\;\times \;88}{100}=73\%$$ (6)

Since pretreatment liquids contain substances that inhibit enzyme activity, it is important to assess the level of the inhibitory effect. For doing that, analytical enzymatic saccharification is run in parallel for microcrystalline cellulose suspended in either pretreatment liquid or a buffer solution. A parameter showing the degree of inhibition (DI) (Eq. 7) can be calculated by comparing the enzymatic digestibility of both reactions:

$$DI\;\left(\%\right)=\frac{\;ED_{Buffer}-ED_{Pret\;liq}}{ED_{Buffer}}\times 100$$ (7)

This calculation procedure can also be applied if it is necessary to assess feedback inhibition by sugars released during pretreatment and contained in the pretreatment liquid. In such a case (Wang et al., 2018b), references with the relevant sugars are included in the enzymatic saccharification experiment.

### Fermentability

Hydrothermal pretreatment of lignocelluloses generates by-products that are inhibitory to fermenting microbes such as yeasts or bacteria. The toxic effects of inhibitory by-products on fermenting microbes (most commonly *S. cerevisiae*) are evaluated through the fermentability of pretreatment liquids. Fermentability is analyzed by carrying out microbial fermentation in the pretreatment liquids (PL) fortified with sugar and nutrient mixtures, and a control fermentation medium devoid of PL can be used as the reference (Martín et al., 2002). Using ethanolic fermentation as an example, important indicators include: the ethanol yield on consumed sugar (Y_con_), the ethanol yield on initial sugar (Y_ini_), the volumetric ethanol productivity (Q), and the specific ethanol productivity (q). Y_con_ is calculated as the amount of ethanol formed per gram of consumed sugar, and Y_ini_ is calculated as amount of ethanol formed per gram of initial sugar content. Q is calculated as the amount of ethanol produced per liter of culture medium and hour during the fermentation [g/(L/h^−1^)]. Q is dependent on the inoculum size and it can be enhanced by increasing the inoculum size and by performing fermentations at high cell density. The specific ethanol productivity, q, is indicative of the performance of a microbial strain. It is often calculated as the volumetric productivity divided by the initial cell concentration [g/(g/h^−1^)].

## Conclusion

Technologies for biochemical conversion of biomass through a sugar-platform approach including hydrothermal pretreatment and enzymatic saccharification have developed rapidly during recent decades and reached industrial implementation. Despite this, intensive research efforts continue and further technology improvements can be expected in the near future. These will likely include innovative technologies addressing aspects such as conditioning to improve biocatalytic performance, enzyme properties and enzyme recycling, microbial strain development, carbon capture and storage with regard to both heat and power generation and carbon dioxide from fermentation processes, lignin valorization, and characterization and management of residual streams, such as pretreatment vapors and stillage constituents.
